# Joint Hypermobility Syndrome and Membrane Proteins: A Comprehensive Review

**DOI:** 10.3390/biom14040472

**Published:** 2024-04-12

**Authors:** Raquel Pliego-Arreaga, Juan Antonio Cervantes-Montelongo, Guillermo Antonio Silva-Martínez, Fabiola Estefanía Tristán-Flores, Miguel Angel Pantoja-Hernández, Juan Raúl Maldonado-Coronado

**Affiliations:** 1 Escuela de Medicina, Universidad de Celaya, Celaya 38080, Guanajuato, Mexico; antonio.cervantes@itcelaya.edu.mx (J.A.C.-M.); mpantoja@udec.edu.mx (M.A.P.-H.); juan.maldonado@udec.edu.mx (J.R.M.-C.); 2 Departamento de Ingeniería Bioquímica, Tecnológico Nacional de México en Celaya, Celaya 38010, Guanajuato, Mexico; guillermo.silva@conahcyt.mx; 3 Departamento de Ciencias Básicas, Tecnológico Nacional de México en Celaya, Celaya 38010, Guanajuato, Mexico; fabiola.tristan@itcelaya.edu.mx

**Keywords:** joint hypermobility syndrome, Ehlers–Danlos syndrome, membrane proteins in joint hypermobility

## Abstract

Ehlers–Danlos syndromes (EDSs) constitute a heterogeneous group of connective tissue disorders characterized by joint hypermobility, skin hyperextensibility, and tissue fragility. Asymptomatic EDSs, joint hypermobility without associated syndromes, EDSs, and hypermobility spectrum disorders are the commonest phenotypes associated with joint hypermobility. Joint hypermobility syndrome (JHS) is a connective tissue disorder characterized by extreme flexibility of the joints, along with pain and other symptoms. JHS can be a sign of a more serious underlying genetic condition, such as EDS, which affects the cartilage, bone, fat, and blood. The exact cause of JHS could be related to genetic changes in the proteins that add flexibility and strength to the joints, ligaments, and tendons, such as collagen. Membrane proteins are a class of proteins embedded in the cell membrane and play a crucial role in cell signaling, transport, and adhesion. Dysregulated membrane proteins have been implicated in a variety of diseases, including cancer, cardiovascular disease, and neurological disorders; recent studies have suggested that membrane proteins may also play a role in the pathogenesis of JHS. This article presents an exploration of the causative factors contributing to musculoskeletal pain in individuals with hypermobility, based on research findings. It aims to provide an understanding of JHS and its association with membrane proteins, addressing the clinical manifestations, pathogenesis, diagnosis, and management of JHS.

## 1. Introduction to Joint Hypermobility Syndrome (JHS) and Its Clinical Manifestations

Joint hypermobility syndrome (JHS), also recognized as hypermobile Ehlers–Danlos syndrome (EDS) or benign joint hypermobility syndrome (BJHS), denotes a connective tissue disorder marked by excessive joint mobility beyond the typical range due to connective tissue laxity, slight changes in skin appearance, and pain of fluctuating intensity [1,2,3,4]. This condition predominantly affects the musculoskeletal system, presenting with a spectrum of clinical manifestations that often pose diagnostic and management challenges. JHS prevalence exhibits wide variation among different populations, with estimates ranging from 10% to 30% in various studies, contingent upon diagnostic criteria and studied cohort demographics [5,6]. In up to 50% of cases, first-degree relatives with the disorder can be identified. Additionally, many patients with joint hypermobility display supplementary signs and symptoms, assisting in distinguishing between various connective tissue disorders, including Marfan syndrome, Stickler syndrome, or most types of EDS [7]. JHS as EDS is the most common type of heritable connective tissue disorder (HCTD) [8,9].

JHS presents as a condition marked by widespread joint hypermobility and various connective tissue manifestations affecting diverse bodily structures, including the skin, eyes, bones, and internal organs [10]. Clinically, individuals with JHS commonly experience generalized joint laxity, frequently accompanied by joint pain, musculoskeletal complaints, and a predisposition to joint dislocations or subluxations [11]. This laxity originates from inherent collagen and extracellular matrix (ECM) abnormalities, resulting in compromised structural integrity of ligaments, tendons, and other supportive tissues surrounding the joints [12,13]. In more severe cases, even minor physical injuries can result in significant damage to bones, tendons, ligaments, muscles, and skin, as well as impaired wound healing [11,14]. Patients at the extreme end of the hypermobility spectrum might also experience systemic symptoms related to fragile connective tissues, including joint and myofascial pain, gastrointestinal issues, postural orthostatic tachycardia syndrome, mast cell activation disorders, and the psychological challenges associated with coping with these limitations [15]. Differences between distinct types of hypermobility syndromes lie in their molecular basis, linked to mutations in ECM proteins genes and enzymes involved in the processing and assembling of ECM proteins [16].

The occurrence of joint hypermobility among individuals with rheumatoid arthritis can indicate a progression of the disease, highlighting the development of common joint issues and local manifestations [17]. The data indicate that individuals with EDS who experience back pain, joint pain, and joint laxity are more likely (67.1%) to receive a diagnosis of at least one rheumatological condition when they undergo a thorough serological and radiographic assessment for their musculoskeletal issues, alongside a physical examination [18]. In recent years, a clinical case definition has emerged for fibromyalgia syndrome with hypermobility developing after a COVID-19 infection. The overlap between JHS and fibromyalgia is 80%, yet the explanation remains unknown. Symptoms include pain after exercise and fibromyalgia starting from a single tendon, suggesting connective tissue involvement in hypermobile individuals. COVID-19 may trigger immunologic responses to connective tissue proteins, leading to fibromyalgia syndrome [19].

Diagnosing JHS involves a comprehensive assessment integrating clinical evaluation, joint mobility scoring systems (like the Beighton score), and, in select cases, genetic testing to exclude other hereditary connective tissue disorders [15]. Besides these points, nowadays, attempts to diagnose this syndrome are based on the 2017 diagnostic criteria [8,19,20], since no instrumental, histopathological, structural, or molecular information is currently available to be used as a specific biomarker [21,22]. However, some patients whose signs and symptoms do not fit completely with the 2017 diagnostic criteria are not officially recognized by all healthcare systems and are categorized only as having hypermobility syndrome disorder (HSD) [23,24].

Comprehending the intricate relationship between joint laxity and the diverse spectrum of clinical manifestations in JHS holds pivotal importance for enhancing diagnostic precision, optimizing patient care, and exploring targeted therapeutic interventions to alleviate symptoms and augment the quality of life for affected individuals.

## 2. Etiologies of Musculoskeletal Pain and Hypermobility

Hypermobility is a condition characterized by an exceptional range of joint motion that exceeds typical limits. While advantageous in activities like gymnastics or dance, hypermobility can also lead to musculoskeletal discomfort and associated symptoms. BJHS denotes a condition marked by joint hypermobility, musculoskeletal pain, and related symptoms in the absence of any other underlying medical condition. It is a prevalent cause of musculoskeletal pain among children and adolescents. Estimates of BJHS prevalence in the general population hover around 10–15%, although this varies with diagnostic criteria. The precise etiology of BJHS remains somewhat elusive, posited as a multifactorial condition influenced by both genetic and environmental elements. Several genetic mutations, including those in the COL1A1 and COL3A1 genes governing collagen types I and III, respectively, have been linked to BJHS [25].

Environmental factors, such as physical activity, also play a role in BJHS development. A study in *The Journal of Rheumatology* highlighted that children engaged in high-impact sports, like gymnastics or dance, were more prone to hypermobility and musculoskeletal pain than their non-participating peers. The repetitive stress from these activities leads to microtrauma and inflammation, exacerbating joint laxity and discomfort. EDS constitutes a cluster of genetic disorders characterized by joint hypermobility, skin hyperextensibility, and other connective tissue anomalies. EDS arises from mutations in genes responsible for collagen or other connective tissue protein synthesis and maintenance [26]. The various EDS subtypes exhibit distinct clinical features and genetic mutations.

Joint hypermobility represents a common trait across several EDS subtypes, notably the hypermobility type (formerly EDS type III). In EDS, joint hypermobility tends to be more severe than in BJHS and can result in joint dislocations and complications. Musculoskeletal pain, often stemming from joint instability, soft tissue injuries, or neuropathic sources, is a prevalent feature [27].

Marfan Syndrome, a genetic disorder marked by connective tissue irregularities, encompasses joint hypermobility, skeletal deformities, and cardiovascular abnormalities. The syndrome originates from mutations in the FBN1 gene, responsible for fibrillin-1 synthesis, a protein crucial in the maintenance of elastic fibers within connective tissues. Joint hypermobility is a typical feature of Marfan syndrome, leading to dislocations and associated musculoskeletal pain. Cardiovascular abnormalities, including aortic aneurysms, can also contribute to chest pain and related symptoms.

Several other genetic disorders, such as Stickler syndrome, osteogenesis imperfecta, and Loeys–Dietz syndrome, manifest with joint hypermobility and musculoskeletal pain due to mutations affecting collagen or proteins crucial in connective tissue synthesis and maintenance [25].

Beyond genetic factors, non-genetic influences contribute to joint hypermobility and musculoskeletal discomfort, including physical activity. High-impact sports and repetitive or poorly executed activities can exacerbate these issues. Poor posture can also lead to muscle imbalances and joint hypermobility, especially in the spine and pelvis. Acute traumas, if inadequately treated, may result in joint hypermobility and related pain. Furthermore, certain neuromuscular disorders, like cerebral palsy or muscular dystrophy, may induce joint hypermobility and musculoskeletal discomfort due to muscle weakness or spasticity [25]. Musculoskeletal pain linked to hypermobility stems from various sources, encompassing genetic disorders, physical activity, postural irregularities, trauma, and neuromuscular conditions. A comprehensive clinical assessment, integrating a detailed medical history, physical examination, and appropriate diagnostic tests, remains crucial in identifying underlying causes. Treatment options span from physical therapy to pain management, and in certain cases, surgical intervention. Early identification and proper management of hypermobility-related musculoskeletal pain significantly enhance quality of life and avert long-term complications.

## 3. Diagnostic Criteria and Challenges in Diagnosing JHS

Diagnostic criteria for BJHS necessitate hypermobility in at least four joints without any concurrent medical condition, as indicated by a review article in *Pediatric Rheumatology* [26]. Despite its prevalence, current diagnostic criteria for JHS lack specificity, posing challenges in differentiating it from familial articular hypermobility and EDS. As a result, there is a critical need to accurately diagnose the underlying disorder responsible for joint hypermobility, which often manifests gradually over time [28].

The Beighton scoring system stands as a widely employed assessment tool to gauge joint flexibility, commonly utilized in the evaluation of JHS [29]. However, it is crucial to note that some individuals with JHS may not exhibit overt symptoms [30]. This underscores the importance of comprehensive studies encompassing significant cohorts affected by joint hypermobility, aiming to unravel the intricate interplay between initial clinical characteristics and subsequent adverse outcomes [28].

Primary therapeutic approaches for managing JHS revolve around bolstering muscle strength and overall fitness to provide enhanced protection for vulnerable joints. Physical modalities such as physiotherapy, occupational therapy, and podiatry assume pivotal roles in mitigating pain levels and diminishing the likelihood of joint dislocations [29,30]. This highlights the necessity for future research to focus on this approach to better understand the multifaceted nature of JHS, the challenges in its diagnosis, the significance of comprehensive studies, and the pivotal role of targeted therapies in managing its manifestations.

## 4. Proteome Profiling of Membrane Proteins as a Diagnostic Tool for JHS

Membrane proteins, a class of proteins embedded in the cell membrane, play a crucial role in cell signaling, transport, and adhesion. Dysregulated membrane proteins have been implicated in a variety of diseases, including cancer, cardiovascular disease, and neurological disorders; recent studies have suggested that membrane proteins may also play a role in the pathogenesis of JHS.

In this context, Chiarelli et al. conducted a comprehensive transcriptome-wide expression profiling analysis using five skin fibroblast strains isolated from adult patients exhibiting full-blown characteristics of JHS/EDS-HT. Their research revealed a consistent alteration in multiple structural proteins of the ECM [31].

Connective tissues comprise an ECM with a specific composition generated during embryogenesis and maintained in adult life. The ECM is a complex network that provides a structural scaffold to surrounding cells and is a reservoir of bioactive molecules such as cytokines and growth factors that control cellular behavior. The main components of the ECM include proteoglycans, hyaluronic acid, adhesive glycoproteins such as fibronectin and laminins, and fibrous proteins such as collagen and elastin. Matricellular proteins like thrombospondins, osteopontin, periostin, and tenascins are nonstructural ECM proteins primarily mediating cell–matrix interactions. They are abundantly expressed during embryonic development, wound healing, and tissue renewal [32].

In addition to providing essential physical support and structural integrity, the precise composition and organization of the ECM play a pivotal role in maintaining cellular health. The ECM experiences continuous turnover, whether under physiological conditions or in pathological circumstances, emphasizing the critical importance of its homeostasis for the architecture and function of connective tissues. Specific cell surface receptors known as integrins facilitate intricate cell–matrix interactions. Comprising heterodimeric transmembrane receptors with α and β subunits, these bridging molecules establish connections between the ECM and the cytoskeleton. Through interactions with collagens and other matrix molecules in their extracellular domain and with cytoskeletal components (e.g., actin, vinculin, talin, paxillin) in their cytoplasmic tails, integrins mediate cell adhesion and motility [32].

The integrin profile observed in EDS/HSD reveals a downregulation of the collagen receptor, α2β1 integrin, and the fibronectin receptor, α5β1 integrin, accompanied by an upregulation of the vitronectin receptor, αvβ3 integrin. Notably, in EDS/HSD, the αvβ3 integrin serves as an alternative receptor for fibronectin. This distinctive integrin expression pattern is coined the ‘integrin switch’, a phenomenon replicated in control cells through functional blocking of the α2β1 receptor. This experiment underscores that the absence of a structurally organized collagen in the ECM, preventing engagement with its receptor α2β1, results in the altered integrin expression profile. Consequently, this shift is poised to influence various cellular processes crucial in the pathogenesis of EDS/HSD [31].

In addition, due to the pivotal role of integrins in mediating cellular responses, intracellular signaling pathways may also be activated by the cadherin superfamily. Comprising calcium-dependent transmembrane proteins, cadherins form intricate adhesions and establish connections with the actin cytoskeleton through a network of associated proteins. Notably, transcriptome profiling of EDS/HSD cells has unveiled a differential expression of numerous genes encoding adhesion molecules. This includes members of the cadherin and protocadherin families, such as CDH2, CDH10, PCDH9, PCDHB16, and PCDHB18, as well as claudins (CLDN11) and desmosomes (desmoplakin, DSP). These molecules play vital roles in the formation of specialized cell–cell junction complexes, which are crucial for maintaining epithelial integrity, orchestrating morphogenesis, and preserving tissue architecture [32].

Chiarelli et al. have unequivocally validated the phenotypic transformation of EDS/HSD fibroblasts into migrating myofibroblast-like cells in their in vitro investigations. This transition is characterized by the expression of hallmark markers, including α-SMA and cadherin-11, heightened levels of the matrix metalloproteinase 9 (MMP9), and an altered expression profile of inflammation mediators CYR61 and CTGF. A signal transduction pathway initiated by the αvβ3 integrin, which signals through the integrin-linked kinase (ILK) and the transcription factor Snail1, orchestrates the induction of this phenotypic switch. Notably, the myofibroblast-like phenotype observed in vitro appears to mirror a persistent in vivo inflammatory-like condition, aligning with the systemic clinical manifestations seen in patients. These manifestations encompass gastrointestinal dysfunction, increased susceptibility to osteoarthritis, chronic generalized musculoskeletal pain, inflammatory soft-tissue lesions, and neurological features [32].

An alternative study [33] revealed that dermal fibroblasts in EDS exhibit a distinct gene expression profile compared to control cells. Notably, these differentially expressed genes are associated with ECM organization, cell–cell and cell–ECM interactions, as well as processes related to pain and inflammatory responses. They identified dysregulation in calcium-related proteins, specifically CALM1 and CALM2. These proteins operate as intracellular calcium signaling sensors, orchestrating the activation of calcium-/calmodulin-dependent protein kinases. This activation, in turn, regulates various crucial processes, including cytoskeleton remodeling, cellular metabolism, differentiation, and proliferation. Furthermore, they noted differential expressions in several members of the annexin family, renowned for their roles in signaling, proliferation, and inflammation [33].

These findings propose that the commonality and defining characteristics among EDS/HSD subtypes may not lie in inherent defects in the structure or organization of collagen itself but rather in the adhesion of cells to collagen. If this hypothesis holds true, it could serve as a foundation for the development of diagnostic tools tailored to discern distinct EDS/HSD subtypes [34].

Owing to their distinctive characteristics, membrane proteins exhibit significant potential as biomarkers. Numerous studies [35,36] emphasize the critical role of proteome profiling of membrane proteins as a diagnostic tool. These investigations underscore the importance of discerning the proteomic landscape of membrane proteins for diagnostic purposes.

In the early stages of membrane protein identification, endeavors were centered on forecasting their identity, structure, and function through the utilization of bioinformatics tools, relying on gene sequences. Nonetheless, it became evident that the linear nucleic acid sequence within the gene does not exhibit a direct correlation with protein expression, structural attributes, or function. Furthermore, it is crucial to recognize that not all membrane proteins are exclusively localized on the cell surface [35].

In another effort to characterize membrane proteins, mRNA-based strategy was employed. This method revolves around the subcellular localization of translation, wherein the synthesis of proteins from mRNA is crucial for the identification of this protein subset. The encoding of membrane proteins occurs through the translation of mRNAs by polyribosomes attached to the cytoplasmic surface of the endoplasmic reticulum. This spatial specificity facilitates the segregation of proteins into distinct fractions—free and membrane-associated mRNA—through equilibrium density gradient centrifugation. Subsequently, each mRNA fraction is differentially labeled with a fluorescent dye and then analyzed using cDNA microarrays to delineate the encoded proteins. To validate the efficacy of this approach, the obtained results were rigorously compared with data derived from in silico algorithms and carefully selected published datasets. This comprehensive validation process ensures the reliability and accuracy of the identified membrane proteins, establishing confidence in the outcomes of this mRNA-based investigative methodology [35].

Both methodologies (gene sequences and mRNA-based strategies) have been adeptly employed to discern differentially expressed proteins, providing promising diagnostic prospects. Some of tools for the precise detection of these proteins are the following: multiplexed immunohistochemistry/immunofluorescence, high-throughput flow cytometry, bottom–up mass spectrometry, top–down mass spectrometry, mass cytometry, Cell-SELEX, and affinity-based isolation, among others. The strategic integration of these techniques not only enriches our understanding of the molecular landscape but also paves the way for establishing diagnostic modalities to decipher the pathogenesis of JHS [35,36].

## 5. Cellular and Molecular Mechanisms Involved in the Pathogenesis of JHS, with a Focus on Membrane Proteins

Collagen, a fundamental element within the extracellular matrix (ECM), plays a crucial role in maintaining tissue integrity and functionality. Mutations occurring in collagen genes, notably types III and V, are significant contributors to the development of Ehlers–Danlos syndrome (EDS). Furthermore, the functions of other ECM constituents, such as tenascin XB (TNXB), vitronectin (VTN), and proteoglycans and glycosaminoglycans (GAGs), are explored within the context of regulating connective tissue equilibrium and understanding pathogenesis [21,22,23,24,25,26,27,28,29,30,31,32,33,34,35,36]. Acquiring insights into these mechanisms is imperative for the advancement of targeted therapeutic interventions and enhancing the clinical management of connective tissue disorders [37,38,39,40].

### 5.1. Collagen

EDS is a heritable disorder of connective tissue, with the type III variety distinguished by joint hypermobility and minor hyperextensibility and softness of the skin. Additionally, the collagen fibril structure has been demonstrated to be abnormal in individuals with this condition. Collagen, pivotal in the ECM of connective tissues like tendons, ligaments, and cartilage, undergoes alterations due to these mutations, resulting in weakened and more elastic connective tissues. This contributes to joint hypermobility and subsequent musculoskeletal discomfort.

**Figure 1:**
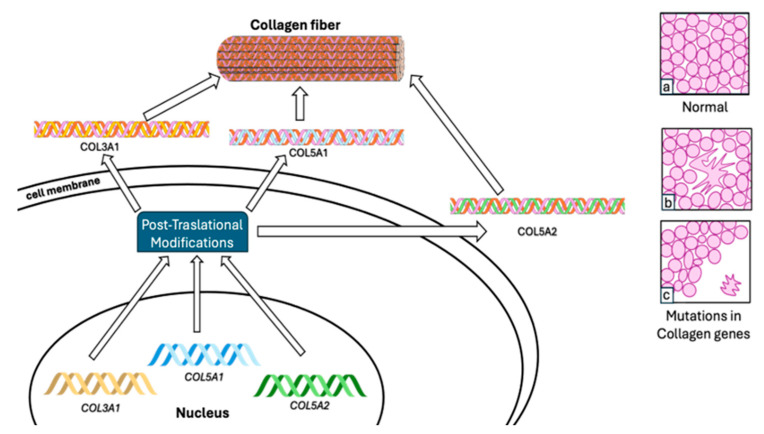
Mutations in collagen genes and their impact on collagen fibrils: Mutations identified in collagen genes such as COL3A1, COL5A1, and COL5A2 have been associated with JHS. These mutations impact the structure of collagen fibrils, changing from a normal distribution and shape (**a**) to abnormal morphologies: cauliflower-like (**b**) or acquiring a variable diameter and irregular spacing (**c**).

In joint hypermobility syndrome/Ehlers–Danlos syndrome hypermobility type (JHS/EDS-HT), mutations in type III collagen can lead to either EDS IV or EDS II [38]. A mutation in collagen type III (COL3A1) has been documented in JHS/EDS-HT, resulting in the severe vascular type of EDS (MIM 130050) [15]. Several mutations in type V collagen have been discovered in JHS/EDS-HT, including COL5A1 and COL5A2 (Figure 1). In addition to mutations in types I and III collagen (COL1A1, COL1A2, and COL3A1), these mutations exhibit an autosomal dominant inheritance pattern in JHS/EDS-HT [23,24,25,26]. Approximately fifty percent of point mutations are observed within the glycine residues of the G-X-Y tripeptide unit, constituting the repetitive sequence of the triple helix [41,42]. Substitution mutations are thought to detrimentally affect collagen function by introducing larger side chains in comparison to glycine. This can lead to localized interference with the folding of the triple helix, a phenomenon associated with joint hypermobility [43].

The collagen family comprises 28 members, expressed by 43 genes, and characterized by the shared triple helix motif. Collagens are categorized into various families, such as membrane (type XVII), network (IV), fibrillar (types I, II, III, V, XI, and XIV), and others (types VII and XXVIII) [44]. Membrane collagens exist in two distinct forms: a transmembrane form and a soluble form released through shedding, which regulates cell behavior [45]. Excessive collagen deposition occurs in the ECM during fibrosis, and fibrillogenesis emerges as a novel target to mitigate fibrosis by disrupting telopeptide-mediated interactions among collagen molecules [46]. Collagens engage with cells through multiple receptors, and their involvement in regulating cell growth, differentiation, and migration via receptor binding is extensively documented [47]. The ECM incorporates additional collagens that actively participate in cell–matrix interactions via diverse receptor families [48,49,50]. A structurally and functionally diverse group of cell surface receptors facilitates the recognition of triple-helical collagen, including integrins, discoidin domain receptors, glycoprotein VI, leukocyte-associated Ig-like receptor-1, and members of the mannose receptor family [51].

The diagnosis of the kyphoscoliotic type of Ehlers–Danlos syndrome (kEDS) represents a rare HCTD characterized by a deficiency in collagen lysyl hydroxylase 1 (LH1) due to mutations in PLOD1. This deficiency leads to the under-hydroxylation of collagen lysyl residues, resulting in an abnormal pattern of lysyl pyridinoline (LP) and hydroxylysyl pyridinoline (HP) crosslinks excreted in the urine. The diagnostic assessment involves evaluating the activity of the lysyl hydroxylase enzyme in skin fibroblasts [52].

### 5.2. Tenascin XB

TNX is an ECM protein with the potential to influence connective tissue in its diverse forms. Its mRNA is notably expressed in fetal muscle and testis, with lower levels in the fetal adrenal gland, kidney, and lung. Furthermore, TNX belongs to a family of ECM glycoproteins that share a common general structure [53]. Initially identified due to its 3′overlap with the CYP21B gene, CYP21B encodes steroid 21-hydroxylase, an essential enzyme in cortisol and mineralocorticoid biosynthesis. Deficiency of this enzyme leads to the recessive disorder known as congenital adrenal hyperplasia [54]. TNX is also known for its capability to bind decorin, a small, leucine-rich proteoglycan that avidly binds collagen and regulates fibrillogenesis. TNX interacts directly or indirectly with multiple fibrils [55,56]. JHS/EDS-HT exhibits significant overlap with a small subset of patients with homozygous/compound heterozygous TNXB mutations.

Identifying JHS/EDS-HT presents challenges due to the absence of specific physical findings and clearly identified causative gene(s). Only a very limited number of cases, still under debate, exhibit mutations in tenascin XB (TNXB) and collagen type III 1 genes [15,19,31]. Zweers et al. [37] reported that haploinsufficiency of an ECM molecule, tenascin-X (TNX), is associated with JHS/EDS-HT. Among the 20 heterozygous individuals displaying TNX haploinsufficiency, irrespective of clinical symptoms, 45% (9 individuals) presented with generalized joint hypermobility, recurrent joint dislocations, and chronic joint pain—symptoms akin to those observed in patients with JHS/EDS-HT. Additionally, 7.5% (6 individuals) out of 80 patients with EDS-HT exhibited haploinsufficiency of TNX, marked by significantly reduced serum TNX levels [37]. Observations indicate that haploinsufficiency of TNXB displays incomplete penetrance in males and partial penetrance in females [37]. Additionally, Schalkwijk reported the presence of TNX in the serum of all subjects without these conditions, individuals with psoriasis, those with rheumatoid arthritis, and 146 out of 151 patients with EDS [50]. Patients with a complete deficiency of TNX displayed notable joint hypermobility, skin hyperextensibility, and easy bruising. TNX-deficient patients exhibited fibrils with a normal appearance and reduced collagen density content. However, irregular and immature elastin fibers and microfibrils were observed in the dermis [51]. In mice, an examination of TNX-null skin compared to wildtype littermates revealed relatively normal size and shape of collagen fibrils. the dermis exhibited a notable decrease in fibril density, ultimately resulting in a 30% reduction in skin collagen content [52]. It also shares similarities with heterozygous carriers of mutations leading to TNXB haploinsufficiency, including a recurrent 30 kb deletion involving TNXB and CYP21A2 [37,50].

### 5.3. Vitronectin

Vitronectin (VTN), also known as “epibolin” and “S protein” [57], is an extracellular matrix glycoprotein with three glycosylation sites, accounting for approximately 30% of its molecular mass [58]. VTN plays a pivotal role as the master regulator of the extracellular environment [59]. Present in plasma, it exists in two forms: a single-stranded 75 kDa polypeptide and a two-stranded form consisting of 65 kDa and 10 kDa polypeptides linked by a disulfide bond [60]. VTN comprises distinct domains, including the SMB domain, the hemopexin-rich region (HPX), the cell attachment site, and the heparin-binding domain. Notably, amino acid residues 45–47 harbor the ARG-GLY-ASP (RGD) motif, serving as the binding site for integrin receptors [61]. VTN typically interacts with cells via integrins on the cell membrane rather than through direct contact. Integrins, a family of cell adhesion receptors, facilitate the connection between the extracellular matrix (ECM) and both the cell membrane and the cytoskeleton [62]. VTN has been identified as a protein with differential levels in the sera of patients with JHS compared to control individuals [38]. VTN is a multifunctional glycoprotein found in plasma, playing roles in cell migration, blood coagulation, fibrinogenesis, the inflammatory process, and membrane attack complex (MAC) formation. VTN directly binds to the nascent precursor C5b-7 complex and C9, featuring distinct binding sites on VTN [63]. As the complement S-protein, VTN occupies the metastable membrane binding site of the nascent precursor complex C5b-7, preventing the newly formed SC5b-7 from inserting into cell membranes. This inhibition of the terminal complement complex (TCC) is thought to occur through charge interactions between the heparin-binding region of VTN and homologous cysteine-rich sequences of the late complement proteins C6, C7, C8, and C9 [63]. The amino-terminal end of vitronectin contains the entire sequence of somatomedin B, followed by an RDG cell attachment sequence and a collagen binding site [64]. VTN has been demonstrated to inhibit C9 binding to the TCC, limiting ongoing membrane-associated pore formation by inhibiting C9 polymerization. This direct influence on lytic pore formation has downstream effects on coagulation, fibrinolysis, and plasminogen activation [58,65]. The proteins of the terminal complement complex (TCC) are water-soluble glycoproteins that constitute two major protein pools in vitro: a membrane-linked complex C5b-9(m) with tissue-damaging potential, and a fluid-phase SC5b-9, which contains an additional regulatory protein—VTN or S-protein (75 kD). It has been proposed that VTN combines with the fluid-phase TCC at the stage of C5b-7 assembly, rendering the subsequently formed SC5b-9 inactive regarding membrane insertion and tissue destruction [66].

### 5.4. Proteoglycan

The main cause of historical Ehlers–Danlos syndromes (EDSs) is the impaired biosynthesis and enzymatic modification of the three main fibrillar collagens. However, EDSs are also linked to abnormalities in proteoglycans, notably in the progeroid and musculocontractural types of EDS, as well as alterations in an endoplasmic reticulum folding protein (observed in EDS with progressive kyphoscoliosis, myopathy, and hearing loss), transcription factors (associated with Brittle cornea types 1 and 2), and a zinc transporter [19,39,67]. Several collagens (IX, XII, XIV, XV, and XVIII) carry glycosaminoglycan chains (such as chondroitin sulfate and/or heparan sulfate chains) and are also considered as proteoglycans [46].

### 5.5. Glycosaminoglycan

Glycosaminoglycans (GAGs) are elongated, unbranched polysaccharides composed of repeating disaccharide units. Through post-translational modifications such as epimerization, sulfation, and acetylation/deacetylation, diverse motifs within the GAG chains are formed, enabling them to bind to a wide range of ligands. Consequently, GAG chains play pivotal roles in regulating growth factor signaling, cell adhesion, proliferation, differentiation, and motility [68].

These glycosaminoglycans extend across collagen fibrils within the ECM of skin and tendons, where the length of the GAG chain determines the width of the interfibrillar gap [69]. The elasticity of the ECM facilitates reversible longitudinal slippage between the antiparallel GAG chains. As tissue stability and elasticity depend on the structure of the GAG bridges, irreversible damage may occur if the bridges lack elasticity [70].

The progeroid type of EDS is attributed to mutations in B4GALT7 or B3GALT6, both of which encode crucial enzymes initiating the synthesis of glycosaminoglycans (GAGs) [71]. The dermatan type of EDS, specifically the D4ST1-deficient type, results from mutations in CHST14, the gene responsible for the post-translational modification of GAGs [39].

### 5.6. Polypetides

C4b-binding protein (C4BP) is composed of two polypeptide chains, α- and β-chains, encoded by separate genes (C4BPA and C4BPB). It serves as a potent circulating soluble inhibitor of the classical and lectin pathways of the complement system and acts as a cofactor for the serine proteinase factor I [72]. During inflammation, C4BPA, expressed at higher levels, binds to C4b, thereby inhibiting the complement activation pathway by reducing the formation and stability of C4bC2b (C3 convertase). Differential levels of the C4BPA protein have been identified in the sera of patients with joint hypermobility syndrome (JHS) compared to control individuals [38].

Notably, studies have shown that the α7β0 isoform of C4BP, lacking the β-chain, induces a semi-mature and anti-inflammatory state in dendritic cells activated by proinflammatory stimuli [73]. Additionally, C4BP serves as an important regulator of the complement system, exhibiting strong binding to apoptotic and necrotic cells, thereby limiting complement activation on these cells [74,75].

## 6. The Relationship between JHS and Ehlers–Danlos Syndromes/Hypermobility Spectrum Disorders

JHS, EDS, and HSD represent a fascinating interplay within the realm of connective tissue disorders, each with its distinctive characteristics, yet intricately connected by the common threads of increased joint flexibility and skin involvement. Their evolution in medical understanding, from JHS being reclassified into the broader spectrum of HSD to the delineation of various EDS subtypes, highlights the intricate web of complexities these conditions encompass [26,76].

Originally identified in the mid-to-late 1960s, JHS, now enveloped within the broader HSD classification, perplexed medical professionals due to its manifestation in otherwise healthy individuals, primarily marked by distressing joint-hypermobility-related pain. This transition in classification, particularly in 2017, marked a pivotal moment by refining the criteria for hypermobile EDS and introducing HSD to encompass diagnoses for those experiencing symptomatic joint hypermobility who did not meet the updated criteria or those for specific syndromes [26,76].

EDSs stand as a constellation of heritable disorders where joint hypermobility and skin involvement serve as unifying factors. However, the presentations diverge significantly among the various EDS types and individuals. Amidst this diversity lies a common culprit: abnormalities in collagen, the fundamental connective tissue scaffold, which impact its structure, production, or processing, ultimately compromising the integrity of connective tissues. HSD, a comprehensive term embracing a spectrum of conditions, mirrors this variability, housing individuals with varying degrees of joint hypermobility and musculoskeletal symptoms. Notably distinct from hypermobile EDS and other syndromes featuring joint hypermobility, individuals with HSD primarily grapple with musculoskeletal manifestations [76]. Diagnosis of these intricate conditions involves a nuanced approach, amalgamating clinical examination, family history analysis, and potentially genetic testing. Evaluation of joint hypermobility utilizing the Beighton score, and a comprehensive questionnaire forms a cornerstone of this diagnostic journey, recognizing diversity in joint flexibility, skin presentations, and associated medical conditions, each exerting varying degrees of impact on daily life [26,77].

The therapeutic landscape for JHS, EDS, and HSD converges around a unified principle—ameliorating symptoms and complications stemming from increased joint flexibility and connective tissue frailty. This holistic approach encompasses tailored strategies, encompassing physical therapy, meticulous pain management, and the collaborative efforts of multidisciplinary teams, delving deep into the multifaceted causes of symptoms [26].

In summary, while JHS, EDSs, and hypermobility spectrum disorders coalesce around joint hypermobility and connective tissue weaknesses, their uniqueness lies in the intricacies of their origins and the varying spectra of severity they present. The reclassification and elucidation of HSD as a distinct category accentuate the imperative of comprehending this spectrum, acknowledging its profound impact on the lives of those affected [26].

## 7. Proteomic Analysis for the Identification of Serum Diagnostic Markers for JHS

Obtaining accurate diagnosis, prognosis, and management is paramount for individuals with hypermobility Ehlers–Danlos syndrome (hEDS); however, there remains a paucity of knowledge regarding a potential reliable biomarker. Given the overlapping features among various EDS variants, the comprehensive differential diagnosis heavily depends on nonspecific clinical aspects. There are some outstanding reports that provide important information for a better understanding of hEDS at a proteomic level with potential extrapolation to be used as a serum biomarker (Table 1) [4,7,37,39,47,48,49,50].

In 2015, Watanabe et al. [38] examined proteins with differential levels in sera from patients with JHS in contrast with control individuals. A total of 106 differentially expressed proteins were identified. The authors centered their attention on six proteins, all of which presented with an increased expression level.

Four of these proteins are involved with the complement system: complement C1r subcomponent (C1R), vitronectin (VTN), complement component C9 (C9), and C4b-binding protein alpha chain (C4BPA). The complement system serves not only in host defense recognition and the elimination of potentially harmful exogenous and endogenous microbial pathogens but also plays a role in various forms of acute and chronic inflammatory diseases such as sepsis and rheumatic diseases. The authors of the study correlate these findings with the possibility of a locally occurring inflammatory process in patients with JHS. However, the potential involvement of the complement system in the development of JHS remains unclear.

Two other proteins of interest are apolipoprotein B-100 (APOB) and transthyretin (TTR). In the context of APOB, the plasma concentration of APOB is recognized as a reliable marker of cardiovascular risk [78]. APOB acts as a ligand for the low-density lipoprotein (LDL) receptor, participating in cholesterol transport to peripheral tissues and its subsequent accumulation in the arterial wall. Additionally, increased levels of apolipoprotein A-I (APOA1) and APOB have been noted in patients with osteoarthritis [79]. On the other hand, TTR (Transthyretin), formerly known as prealbumin, serves as a carrier or transporter for thyroxin and retinol-binding protein (RBP), which specifically transports vitamin A. Under physiological conditions, both TTR and RBP form a macromolecular complex that is thought to have a significant physiological function: preventing glomerular filtration of the low-molecular-weight RBP in the kidneys [80]. There is only one report that associates TTR defects with EDS classical type (cEDS) [81]. At the end, Watanabe et al. concluded that this knowledge will be helpful to establish an early diagnostic method for JHS and possibly contribute to the development of pharmacological therapies.

In 2013, Merke et al. [82] reported the reduced expression of the protein tenascin-X derived from a TNXB haploinsufficiency; this fact has been associated with hEDS [37]. Yamada et al. [83] reported a quantification method for serum TNX (sTNX) levels. In control individuals, 144 ng/mL of sTNX were obtained while sTNX was not detected in patients with cEDS. They conclude that the quantification method will be useful for diagnosis and risk stratification of EDS caused by TNX deficiency and haploinsufficiency. From the same work group, in 2019 [84], the question of whether hEDS could be diagnosed by the concentrations of serum form of TNX (sTNX) was investigated. In half of the hEDS patients that participated in the analysis, the concentration levels of sTNX were significantly lower than those of the control group with a mean concentration of 69.3 ng/mL; meanwhile, in healthy individuals, the mean sTNX concentration in sera was 110 ng/mL. In this study, no association of reduced sTNX concentration and TNXB mutations was found, suggesting that the expression of TNX might be related to epigenetic regulation in hEDS patients. The conclusion mentioned in this research was that the reduced sTNX concentration in hEDS patients could be a risk factor and the need for the verification of this possibility relies on major sample collection and deeper analysis.

Future perspectives have emerged as possible biomarkers; in one interesting report [25], the cellular proteome of hEDS dermal myofibroblasts was compared to that of control fibroblasts, and a total of 183 differentially expressed proteins (DEPs) was obtained. A particularly remarkable example is S100A4, which exhibited elevated levels in hEDS myofibroblasts. This protein plays a fundamental role in various proinflammatory conditions [84,85,86,87,88,89]. S100A4 contributes to the pathological extracellular matrix (ECM) remodeling by modulating the production of different structural components as well as matrix-degrading proteases including matrix metalloproteinases (MMPs) [90,91,92,93,94]. These findings support a possible explanation for the phenomenon seen when control fibroblasts acquire a myofibroblast-like phenotype when treated with hEDS-cell-conditioned medium [95], thus considering S100A4 as a key factor in this process.

Interleukin 6 gene appeared to be downregulated in a functional study of hEDS fibroblasts [96]. It is known that IL6 participates in immune and inflammatory responses and its quantification could be an important biomarker in studying hEDS and its prognosis [97]. Elevated prolactin serum concentration has been related to different pain conditions and inflammation. Since EDS patients experience chronic pain related to hypermobile joints, the hypothesis that increased prolactin levels and EDS is established, but more and deeper analyses need to be carried out to verify this idea [31,97]. Selenium-binding protein-1 (SELENBP1) presents an upregulation expression in blood and brain samples from schizophrenic patients; thus, it is an important candidate as a biomarker for schizophrenia [98]. hEDS patients have been found to develop some psychiatric disorders [99,100]; accordingly, it would be worthwhile analyzing the quantification of this protein in blood as a biomarker to confirm the diagnosis [100].

## 8. Mechanisms and Diagnosis of Folate-Dependent Hypermobility Syndrome

The molecular underpinnings of various types of EDS and hEDS/HSD exhibit notable contrasts. Most EDS subtypes are characterized by mutations in genes responsible for encoding ECM proteins such as collagen or enzymes and chaperones involved in ECM protein processing and assembly [101]. However, the molecular origins of hEDS and HSD remain largely elusive.

**Figure 2:**
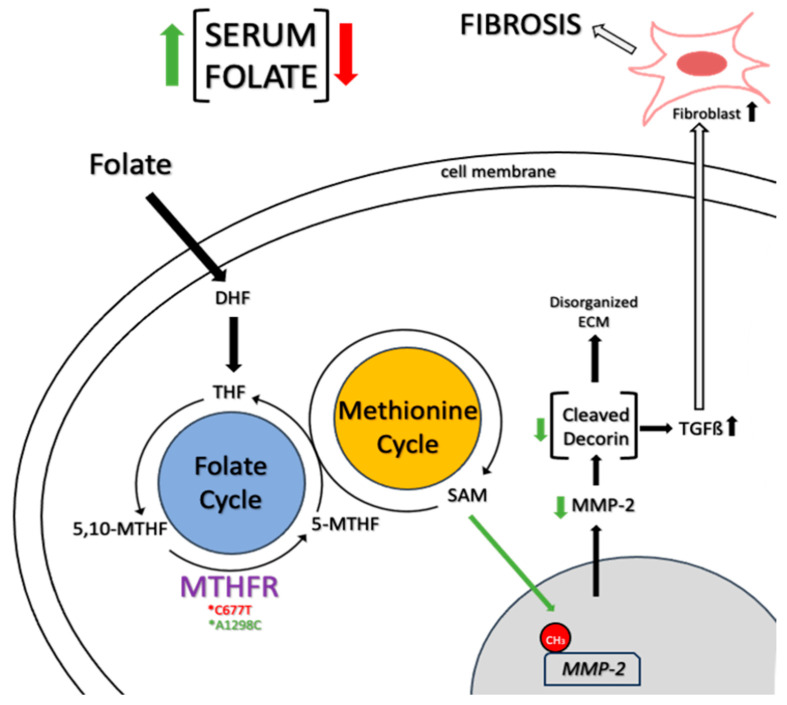
Molecular basis of folate-dependent hypermobility syndrome: MTHFR polymorphisms C677T and A1298C reduce or increase serum folate levels, respectively. High levels of folate increase methylation of the MMP2 gene, leading to low levels of the protein, decreasing cleavage of decorin, disrupting collagen organization, and causing tissue instability and fragility. Additionally, low levels of MMP2 trigger aberrant TGF-ß signaling, initiating fibrotic pathways. Dihydrofolic acid (DHF); Tetrahydrofolate (THF); 5,10-Methylenetetrahydrofolate (5,10-MTHF); Methylenetetrahydrofolate reductase (MTHFR); 5-methyltetrahydrofolate (5-MTHF); S-adenosylmethionine (SAM); Matrix Metalloproteinase 2 (MMP2); Transforming growth factor-β (TGF-β).

While some studies have identified genetic variations associated with hEDS in specific families, and deficiencies in TNX have been linked to hypermobility, these findings are not universal across all cases of hEDS or HSD [10,50]. The lack of clarity concerning the pathophysiology of hypermobility significantly limits the available therapeutic options and support for affected patients. Folate-dependent hypermobility syndrome (FDHS) is characterized by altered folate metabolism (Figure 2)—specifically alterations in the methylenetetrahydrofolate reductase (MTHFR) gene that encodes to a key folate-metabolizing enzyme—which leads to hypermobility symptoms [102,103]. Folates are crucial B9 vitamins involved in various cellular processes. The MTHFR enzyme, responsible for converting 5,10-methylene-tetrahdyrofolate (5,10-MTHF) to 5-methylTHF (5-MTHF), is central to folate metabolism. MTHFR polymorphisms C677T and A1298C, which are heterogeneously distributed worldwide, significantly affect the enzyme function, leading to altered serum folate levels [103,104]. C677T polymorphism correlates with reduced serum folate, while A1298C may elevate folate levels. These polymorphisms are prevalent in approximately 30% of the population and can significantly impact folate metabolism through reduced enzyme function by up to 80% [105,106].

The folate-dependent methylation of histones and DNA plays a vital role in gene expression regulation on different development stages [107]. Altered MTHFR activity could impact the methylation of ECM-modifying enzymes like matrix metalloproteinase 2 (MMP2). MMP2 cleaves decorin, a proteoglycan crucial for tissue integrity, that disrupts collagen organization, leading to tissue instability and fragility [108]. Moreover, this cleavage triggers aberrant transforming growth factor β (TGFβ) signaling, triggering fibrotic pathways and myofascial pain [109,110].

Despite extensive research on folate metabolism, several aspects, especially regarding folate status, remain unclear. Elevated serum folate levels in patients might indicate changes in folate transporters, such as folate receptor α (FRα), which has a high affinity for 5-MTHF and folic acid [111,112]. Autoantibodies blocking FRα could disrupt the transportation of these compounds between intracellular and extracellular compartments. While there is an association between hypermobility and autism spectrum disorder (ASD), there is limited research on the link between MTHFR polymorphisms and mutations in these transporters and receptors. The growing availability of genetic sequencing data might enable future analyses in this regard [113,114].

Potential treatments for changes in folate transportation include supplementation with 5-MTH, the end-product of MTHFR activity, which is transported by reduced folate carrier (RFC) and could potentially mitigate these symptoms by epigenetic stabilization of the MMP2 promoter and the preservation of collagen organization [114,115]. In cases involving FRα autoantibodies, there is evidence in supplementing with 5-MTHF in cerebral folate deficiency and ASD, which has an association with hypermobility, that could facilitate its transportation via FRα more effectively than folic acid. Also, elevated serum folate levels could mask low intracellular folate levels due to the increased conversion of membrane bound FBPs to serum FBPs, potentially affecting study outcomes [113,115,116].

Techniques such as liquid chromatography/mass spectrometry and genetic testing could be employed to analyze serum folate metabolites and mutations related to folate transportation and metabolism. Studying folate receptor autoantibodies and evaluating MMP2 expression and decorin activity in tissue studies could provide valuable insights [114]. Additionally, research on dietary interventions, such as reducing folic acid supplementation, may be useful if folic acid is found to be the specific elevated metabolite in hypermobile patients [115]. Despite the challenges faced by patients with hEDS and HSD and their medical care providers, it is necessary to understand the wide variety of hypermobility symptoms, drawing from clinical observations and the existing literature.

## 9. Conclusions 

The investigation into musculoskeletal pain associated with hypermobility has unveiled multifaceted etiologies, shedding light on the complexities of JHS. Understanding the cellular and molecular mechanisms underlying JHS, particularly the involvement of membrane proteins, stands as a pivotal area of exploration. Research has illuminated aberrations in collagen and extracellular matrix components, yet deeper elucidation of the roles of membrane proteins is vital in comprehending the intricate pathogeneses.

The quest for serum diagnostic markers through proteomic analysis marks a promising avenue, offering potential breakthroughs in JHS diagnosis. Identifying specific biomarkers holds the key to accurate and efficient diagnostic approaches, aiding clinicians in early detection and tailored management strategies.

Moreover, the proposed mechanisms and diagnostic criteria for folate-dependent hypermobility syndrome present an intriguing field for investigation. Further studies are recommended to explore the transportation mechanisms of folate, MTHFR polymorphisms, MMP levels, and decorin’s activity and structure in hypermobile patients. Unraveling the intricate interplay between folate metabolism and joint hypermobility could unlock novel therapeutic avenues and refine diagnostic protocols for this subset of individuals. Nevertheless, diagnosing JHS remains a challenge due to its diverse clinical manifestations and overlaps with other connective tissue disorders. The development of precise and comprehensive diagnostic criteria, coupled with advanced imaging techniques and biomarker identification, will be pivotal in overcoming these diagnostic hurdles. Further research exploring the intricate relationship between JHS and Ehlers–Danlos syndromes/hypermobility spectrum disorders (EDSs/HSDs) stands as an essential future direction. Unraveling the shared and distinctive features between these conditions will refine classification, diagnosis, and targeted therapeutic approaches for affected individuals.

In conclusion, the multifaceted nature of JHSs necessitates continued exploration across various fronts. From elucidating molecular mechanisms to refining diagnostic criteria, development of adequate tools for treatment for affected patients, and exploring intersections with related disorders, ongoing research endeavors hold immense potential to enhance our understanding and management of JHSs.

## Figures and Tables

**Table 1:** Overview of serum biomarker candidates for JHS.

Biomarker Candidates	Expression Landscape	Sample Type	Reference
Proteins involved with the complement system: C1r, C1R, VTN, C14, and C4BPA.	Increased for JHS	Human serum	[4]
Apolipoprotein B-100 (APOB)	Increased for JHS	Human serum	[4,7]
Transthyretin (TTR)	Increased for JHS	Human serum	[4,37]
Protein tenascin-X	Reduced for cEDS and hEDS	Human serum	[4,37]
S100A4	Increased for hEDS	Dermal myofibroblasts	[39,47]
Interleukin 6	Reduced for hEDS	Fibroblasts	[48,49]
Prolactin	Possibly increased for EDS	Fibroblasts	[48,49]
Selenium-binding protein-1 (SELENBP1)	Possibly increased for hEDS and schizophrenia	Blood and brain	[49,50]

JHS, joint hypermobility syndrome; cEDS, Ehlers–Danlos classical type; hEDS, hypermobile Ehlers–Danlos syndrome.
